# Associations of Dietary Iron Intake With Mortality From Cardiovascular Disease: The JACC Study

**DOI:** 10.2188/jea.JE20120006

**Published:** 2012-11-05

**Authors:** Wen Zhang, Hiroyasu Iso, Tetsuya Ohira, Chigusa Date, Naohito Tanabe, Shogo Kikuchi, Akiko Tamakoshi, the JACC Study Group

**Affiliations:** 1 Public Health, Department of Social and Environmental Medicine, Osaka University Graduate School of Medicine, Osaka University, Suita, Osaka, Japan; 1 大阪大学大学院医学研究科社会環境医学講座公衆衛生学; 2 School of Human Science and Environment, University of Hyogo, Himeji, Hyogo, Japan; 2 兵庫県立大学環境人間学部; 3 Department of Human Life, University of Niigata Prefecture, Niigata, Japan; 3 新潟県立大学人間生活学部; 4 Department of Public Health, Aichi Medical University School of Medicine, Nagakute, Aichi, Japan; 4 愛知医科大学医学部公衆衛生

**Keywords:** dietary iron, mortality, stroke, coronary heart disease, cardiovascular disease, follow-up studies

## Abstract

**Background:**

We investigated the relationship between dietary iron intake and mortality from cardiovascular disease (CVD) in a population-based sample of Japanese adults.

**Methods:**

The study cohort consisted of 58 615 healthy Japanese (23 083 men and 35 532 women), aged between 40 and 79 years, who had no history of stroke, coronary heart disease (CHD), or cancer at baseline. Dietary iron intake was assessed at baseline by a validated food frequency questionnaire administered between 1988 and 1990 as part of the Japan Collaborative Cohort (JACC) Study.

**Results:**

We documented 2690 (1343 men and 1347 women) deaths from CVD: 1227 (607 men and 620 women) deaths from total stroke, 651 from ischemic stroke (355 men and 296 women), 459 (196 men and 263 women) from hemorrhagic stroke, and 557 (311 men and 246 women) from CHD. Dietary intake of total iron was positively associated with mortality from total and ischemic stroke and total CVD in men. The multivariable hazard ratio for the highest versus the lowest quintile of total iron intake was 1.43 (95% CI, 1.02–2.00; *P* for trend = 0.009) for total stroke and 1.27 (1.01–1.58; 0.023) for total CVD in men. Dietary total iron intake was not associated with mortality from other endpoints in men, and was not associated with any endpoints in women.

**Conclusions:**

Dietary intake of total iron was positively associated with mortality from stroke and total CVD in Japanese men.

## Abstract (ja)

**[目的]:**

本研究は、日本人を対象とし、大規模縦断研究で鉄の摂取が循環器疾患死亡との関連を研究することを目的とする。

**[方法ならびに成績]:**

本研究は、文部科学省科学研究費がん特定領域大規模コホート研究(JACC Study)の2006年までのデータに基づいて、1988年から1990年のベースライン時に脳卒中、CHDの既往歴がなく、FFQに記入した40-79歳の男性23,083、女性35,532名を研究対象とした。鉄の摂取量について、男女別に残差法によりエネルギーを調整して男女別に五分位で分析を行った。循環器疾患死亡の相対危険度を多変量調整したCox比例ハザードモデルにより算出した。

追跡期間中2,690人（男性1,343人、女性1,347人）、そのうち、脳卒中1,227人（男性607人、女性620人）、脳梗塞651人（男性355人、女性296人)、脳出血459人（男性196人、女性263人),虚血性心疾患557人(男性311人、女性246人)が、循環器疾患で死亡した。男性において、鉄の摂取の最も低い群に対して最高群の多変量調整の全脳卒中と全循環器疾患の死亡リスクはそれぞれ1.43 (95%CI,1.02-2.00, P for trend=0.009)、1.27(95%CI,1.01-1.58, P for trend=0.023)であった。女性において、鉄の摂取と循環器疾患の死亡リスクの間に有意な関連は認められなかった。

**[総括]:**

日本人の男性において、鉄の摂取と全脳卒中および全循環器疾患の死亡リスクの間に正の関連がある。

## INTRODUCTION

Iron is an important mineral for humans because it is responsible for oxygen transport, digestion of food, and metabolism of body fat, which is essential for cell renewal.^1^ However, iron has been implicated in the development of cardiovascular disease because it induces free-radical damage to tissues.^2^ In 1981, Sullivan proposed the “iron hypothesis” (ie, that iron stored in the body increases the risk of cardiovascular disease) after observing myocardial failure in people with iron storage disease, age-related accumulation of stored iron in men, and levels of post-menopausal accumulation of stored iron in women that were similar to those in men.^3^ Although this hypothesis has been tested during the subsequent 30 years, the results have been inconsistent.

Among studies that used serum ferritin as an indicator of body iron stores, a cross-sectional study of German women found a significant positive association between serum ferritin levels and carotid atherosclerosis and noted that this association was more evident among women with higher levels of low-density lipoprotein (LDL) cholesterol.^4^ A prospective study of Italian men and women reported a positive association between serum ferritin level and development of early carotid atherosclerosis, which supports the iron hypothesis.^5^ Another prospective study, of Finnish men, showed that serum ferritin level was positively associated with risk of coronary heart disease,^6^ while a prospective study of Dutch postmenopausal women found a positive association with risk of ischemic stroke.^7^ However, other studies showed nonsignificant relations.^8^^–^^13^ Among studies that used food frequency questionnaires (FFQs) to evaluate dietary iron intake, the prospective study of Finnish men found a positive association between total iron intake and risk of myocardial infarction.^6^ A case-control study of Chinese men and women reported that dietary iron intake was positively associated with risk of ischemic stroke.^14^ Other studies found a positive association between dietary intake of heme iron derived mainly from red meat and risk of coronary heart disease,^15^^,^^16^ whereas others found no, or even inverse, associations.^8^^,^^17^^,^^18^ To our knowledge, no prospective study has examined the association between dietary iron intake and cardiovascular disease incidence or mortality in an Asian population.

Using data from a large prospective study of Japanese men and women, we investigated the association between dietary iron and mortality from cardiovascular disease.

## METHODS

### Study population

The Japan Collaborative Cohort (JACC) Study for the Evaluation of Cancer Risk, a large-scale cohort study sponsored by the Ministry of Education, Sports and Science, was conducted from 1988 to 1990 as a baseline survey. The population-based sample consisted of 110 792 adults (46 465 men and 64 327 women) from 45 community areas. The sampling methods and protocols of the JACC study have been recently described.^19^ Participants were asked to complete self-administered questionnaires concerning their lifestyle behaviors and medical histories of cardiovascular disease and cancer. Subjects were excluded if they reported a history of stroke, coronary heart disease, or cancer (*n* = 5864) at baseline or were unable to provide data for the FFQ (*n* = 46 313). Data from the remaining 58 615 subjects (23 083 men and 35 532 women) were used in the analyses. There were some differences in baseline characteristics between individuals who responded to the FFQ and those who did not, such as mean age (56.5 vs 59.3 years, respectively), college or higher education (12.6% vs 5.7%), and higher perceived mental stress (20.7% vs 7.7%).

Informed consent was obtained either from participants before they completed the questionnaire or from community leaders (which was a common way to obtain informed consent in Japan at the time the study was conducted). The institutional review boards of Nagoya University, University of Tsukuba, and Osaka University approved the present study.

### Assessment of iron intake

Dietary iron intake was assessed at baseline by using a self-administered FFQ, which included 33 food items. Participants were asked to estimate average consumption of certain foods over the previous year. Five frequency responses were provided: rarely, 1 to 2 days per month, 1 to 2 days per week, 3 to 4 days per week, and almost every day. Iron content per 100 g of each food (as determined with the Japan Food Tables, Fifth Revised Edition) and the portion size for each food was estimated in a previous validation study.^20^ Iron intake was calculated from the participants’ frequency scores for the consumption of each food, ie, 0, 0.38, 1.5, 3.5, and 7 times per week. Iron was adjusted for total energy using the residual method. Supplemental iron intake was not measured because we did not have sufficient data on vitamin supplementation. Four 3-day dietary records were collected over a 1-year period (*n* = 85) to test the validity of the FFQ. The records showed that average dietary iron intake was 9.4 mg/day, while that calculated from the FFQ was 6.8 mg/day. The Pearson correlation coefficient between iron intake as estimated by the FFQ and by the four 3-day dietary records was 0.50 after individual energy adjustment. As for reproducibility, 2 questionnaires were administered, 1 year apart, to 85 participants, and the Spearman correlation coefficient was 0.95.

We also used the FFQ to assess heme iron intake. We adopted the method used in the Netherlands Cohort Study on Diet and Cancer (NLCS) and the Canadian National Breast Screening Study (NBSS) database.^21^^,^^22^ The food items that contributed to heme iron intake were beef, pork, sausage, chicken, liver, fish, dried fish, and boiled fish paste. Heme iron was estimated by using the percentage of heme iron to total iron in these major food items, namely, 69% for beef, 39% for pork and ham, 26% for chicken, 21% for liver, and 26% for fish, dried fish, and boiled fish paste. Average daily intake of heme iron was calculated by multiplying frequency of consumption by the amount of total iron and the above percentages. Non-heme iron intake was calculated as total iron minus heme iron intake. Heme and non-heme iron intakes were also adjusted for total energy using the residual method.

### Mortality surveillance

In each community, investigators conducted a systematic review of death certificates as part of the mortality surveillance. Death certificates were forwarded to the public health departments of the respective areas, and mortality data were then centralized at the Ministry of Health and Welfare. Underlying cause of death was coded for National Vital Statistics using the International Classification of Disease, 9th Revision (ICD-9) from 1988 to 1994 and ICD-10 from 1995 to 2003. All deaths within the cohort were ascertained by means of a death certificate from a public health center, except for subjects who died after moving out of their original communities, which were treated as censored cases (*n* = 2956). Follow-up was conducted until the end of 2006, except in 4 areas where it was terminated in 1999 and 4 areas where it was terminated in 2003. The median follow-up period was 14.7 years.

The primary endpoints for this analysis were death from total cardiovascular disease (ICD-9 codes 390–459 and ICD-10 codes 101–199), including total stroke (codes 430–38 and I50–I69)—which was further subdivided into hemorrhagic stroke (430–431 and I60–I61) and ischemic stroke (433–434 and I63)—as well as coronary heart disease (410–414 and I20–I25) and myocardial infarction (410 and I21–I22).

### Data analysis

Data analyses were based on age-adjusted mortality rates for cardiovascular disease endpoints in the 45 study areas during the follow-up period from 1989 to 2006, with the exception of 8 areas (follow-up ended in 1999 in 4 areas and in 2003 in 4 other areas). Duration of follow-up was defined as the period from submission of the initial baseline questionnaire to either death, termination of follow-up, or the departure of a participant from his/her original community. Participants were divided into quintiles of estimated dietary intakes of total, heme, and non-heme iron.

Age-adjusted mean values and proportions of selected CVD risk factors were presented based on the quintiles. We used Cox proportional hazards models to estimate age-adjusted and multivariable-adjusted hazard ratios (HRs) and 95% CIs. We also tested for trends across the quintiles by assigning median values to each quintile. The confounding variables used for adjustment included body mass index (BMI; sex-specific quintiles), smoking status (never, ex-smoker, or current smoker of 1–19 or ≥20 cigarettes per day), ethanol intake (never, ex-drinker, or current drinker of 1–22, 23–45, 46–69, or ≥69 g per day), history of hypertension (yes or no), history of diabetes (yes or no), sports participation time (never, 1–2 hours, 3–4 hours, or ≥5 hours per day), walking time (never, about 30 min, 30–60 min, or ≥60 min per day), educational level (educated until age 12, 13–15, 16–18, or ≥19 years), perceived mental stress (low, medium, or high), and, for women, menopausal status (yes or no) and hormone replacement therapy (yes or no).

All statistical analyses for 2-tailed tests were performed using Statistical Analysis Software (SAS) version 9.13 (SAS Institute Inc., Cary, North Carolina, USA). A *P* value less than 0.05 was considered to indicate statistical significance.

## RESULTS

Tables 1, 2, and 3 show the age-adjusted means and prevalences of cardiovascular risk factors at baseline according to quintiles of dietary total iron, heme iron, and non-heme iron intakes, respectively. The median intake of energy-adjusted total iron was 5.12 mg/day in the bottom quintile and 10.58 mg/day in the top quintile among men; the respective intakes among women were 5.14 mg/day and 9.81 mg/day. The corresponding values of energy-adjusted heme iron intake were 0.07 mg/day and 0.44 mg/day among men and 0.06 mg/day and 0.48 mg/day among women. The corresponding values of energy-adjusted non-heme iron intake were 3.84 mg/day and 10.19 mg/day among men and 3.81 mg/day and 9.46 mg/day among women. The Spearman correlation between total iron intake and heme iron intake was 0.30 for men and 0.28 for women, while the corresponding correlations between total iron intake and non-heme iron intake were 0.73 and 0.70, respectively.

**Table 1.** Baseline characteristics and risk factors of participants by quintile of total iron intake

	Quintile of total iron intake					*P* for trend
	
	1 (low)	2	3	4	5 (high)	
**Men**						
No. of subjects	4616	4617	4617	4617	4616	
Median iron intake (mg/day)^a^	5.12	6.60	7.65	8.78	10.58	
Mean age (SD) (years)	53.4 (9.7)	54.3 (9.6)	55.8 (9.6)	57.1 (9.8)	59.2 (9.8)	<0.001
Mean body mass index (kg/m^2^)	22.8 (2.7)	22.7 (2.7)	22.8 (2.6)	22.6 (2.7)	22.6 (2.8)	0.002
Mean energy intake (SD) (kcal/day)	1688 (492)	1775 (480)	1800 (488)	1783 (493)	1666 (496)	<0.001
Mean dietary sodium intake (mg/day)	1324	1929	2286	2584	2872	<0.001
Current smokers (%)	57.8	52.9	51.7	50.9	47.6	<0.001
Mean alcohol intake (g/day of ethanol)	39.2	36.6	34.0	31.3	27.2	<0.001
History of hypertension (%)	18.5	19.3	19.0	19.2	19.0	0.906
History of diabetes mellitus (%)	6.6	6.2	6.5	5.5	5.9	0.234
Sports time ≥3 hours/week (%)	11.9	12.6	13.2	14.7	16.4	0.050
Walking time ≥30 minutes/day (%)	51.8	48.0	47.1	45.3	44.3	<0.001
College or higher education (%)	16.5	16.7	16.8	18.0	16.7	0.178
High perceived mental stress (%)	26.6	24.5	21.7	20.4	18.4	<0.001
**Women**						
No. of subjects	7106	7107	7106	7107	7106	
Median iron intake (mg/day)^a^	5.14	6.43	7.35	8.31	9.81	
Mean age (SD) (years)	55.0 (10.1)	55.0 (9.7)	55.8 (9.7)	57.1 (9.7)	58.5 (9.7)	<0.001
Mean body mass index (kg/m^2^)	23.0 (3.1)	22.8 (3.0)	22.9 (3.0)	22.9 (3.0)	22.9 (3.0)	0.002
Mean energy intake (SD) (kcal/day)	1415 (403)	1451 (367)	1459 (348)	1466 (340)	1398 (358)	<0.001
Mean dietary sodium intake (mg/day)	1353	1858	2134	2368	2595	<0.001
Current smokers (%)	6.9	4.5	3.8	3.4	3.6	<0.001
Mean alcohol intake (g/day of ethanol)	14.3	9.5	9.2	7.8	7.8	<0.001
History of hypertension (%)	21.0	19.6	20.7	21.1	20.3	0.157
History of diabetes mellitus (%)	3.5	2.9	3.2	3.5	3.9	0.019
Sports time ≥3 hours/week (%)	7.8	8.4	9.3	9.9	11.1	<0.001
Walking time ≥30 minutes/day (%)	47.7	46.3	43.6	44.2	43.5	<0.001
College or higher education (%)	9.0	8.9	10.6	10.2	9.9	<0.001
High perceived mental stress (%)	21.1	20.7	19.8	18.4	16.6	<0.001
Menopause (%)	58.8	60.5	63.2	67.3	70.3	<0.001
Hormone-replacement therapy (%)	4.7	4.6	4.2	4.1	4.5	<0.001

**Table 2.** Baseline characteristics and risk factors of participants by quintile of heme iron intake

	Quintile of heme iron intake					*P* for trend
	
	1 (low)	2	3	4	5 (high)	
**Men**						
No. of subjects	4616	4617	4617	4617	4616	
Median heme iron intake (mg/day)^a^	0.07	0.16	0.22	0.28	0.44	
Mean age (SD) (years)	56.4 (10.1)	55.4 (9.9)	55.4 (9.9)	55.8 (9.8)	56.7 (9.9)	<0.001
Mean body mass index (kg/m^2^)	22.9 (2.8)	22.7 (2.6)	22.7 (2.7)	22.7 (2.7)	22.5 (2.7)	<0.001
Mean energy intake (SD) (kcal/day)	1719 (483)	1768 (485)	1766 (486)	1738 (502)	1722 (506)	<0.001
Current smokers (%)	53.7	51.7	51.8	52.6	53.1	0.054
Mean alcohol intake (g/day of ethanol)	34.2	34.3	33.9	33.8	33.7	0.850
Mean dietary sodium intake (mg/day)	1936	2122	2197	2270	2366	<0.001
History of hypertension (%)	22.1	19.0	17.6	18.7	17.6	<0.001
History of diabetes mellitus (%)	5.0	6.3	5.9	6.5	7.0	0.003
Sports time ≥3 hours/week (%)	11.4	12.5	14.1	14.9	15.9	<0.001
Walking time ≥30 minutes/day (%)	43.0	48.0	48.1	49.8	47.6	<0.001
College or higher education (%)	12.3	16.7	18.5	19.3	17.7	<0.001
High perceived mental stress (%)	19.3	23.0	23.2	24.4	23.4	<0.001
**Women**						
No. of subjects	7106	7107	7106	7107	7106	
Median heme iron intake (mg/day)	0.06	0.16	0.21	0.28	0.48	
Mean age (SD) (years)	57.7 (10.0)	56.5 (10.0)	55.9 (9.8)	55.5 (9.6)	55.7 (9.8)	<0.001
Mean body mass index (kg/m^2^)	23.0 (3.1)	22.8 (3.0)	22.8 (2.9)	22.8 (3.0)	22.8 (3.0)	0.009
Mean energy intake (SD) (kcal/day)	1410 (373)	1470 (358)	1458 (355)	1441 (355)	1410 (378)	<0.001
Mean dietary sodium intake (mg/day)	1873	2021	2073	2107	2128	<0.001
Current smokers (%)	5.2	4.2	3.9	4.2	4.5	0.001
Mean alcohol intake (g/day of ethanol)	11.6	11.1	9.8	9.0	9.5	<0.001
History of hypertension (%)	25.7	21.3	19.2	18.8	17.6	<0.001
History of diabetes mellitus (%)	3.7	3.3	3.1	3.5	3.4	0.605
Sports time ≥3 hours/week (%)	8.0	9.6	9.2	9.4	10.3	<0.001
Walking time ≥30 minutes/day (%)	41.2	45.3	46.3	46.4	46.2	<0.001
College or higher education (%)	5.9	9.8	10.0	11.8	11.2	<0.001
High perceived mental stress (%)	17.4	18.9	20.2	20.5	19.5	<0.001
Menopause (%)	68.2	64.3	63.3	62.0	62.2	<0.001
Hormone-replacement therapy (%)	4.0	4.0	4.3	5.2	4.5	<0.001

**Table 3.** Baseline characteristics and risk factors of participants by quintile of non-heme iron intake

	Quintile of non-heme iron intake					*P* for trend
	
	1 (low)	2	3	4	5 (high)	
**Men**						
No. of subjects	4616	4617	4617	4617	4616	
Median non-heme iron intake (mg/day)	3.84	6.05	7.23	8.40	10.19	
Mean age (SD) (years)	54.9 (10.1)	54.0 (9.6)	55.1 (9.6)	56.7 (9.7)	58.1 (9.7)	<0.001
Mean body mass index (kg/m^2^)	22.9 (2.7)	22.7 (2.7)	22.7 (2.6)	22.6 (2.7)	22.6 (2.8)	0.002
Mean energy intake (SD) (kcal/day)	1697 (491)	1754 (484)	1796 (488)	1795 (493)	1671 (494)	<0.001
Mean dietary sodium intake (mg/day)	1655	1756	2188	2537	2860	<0.001
Current smokers (%)	56.4	54.6	51.2	51.2	47.4	<0.001
Mean alcohol intake (g/day of ethanol)	37.7	36.9	34.9	31.8	27.2	<0.001
History of hypertension (%)	20.6	18.7	18.5	18.5	18.7	0.053
History of diabetes mellitus (%)	6.2	5.5	6.6	5.7	6.5	0.075
Sports time ≥3 hours/week (%)	12.3	12.3	13.2	14.8	16.1	0.033
Walking time ≥30 minutes/day (%)	46.0	50.5	48.8	45.3	45.0	<0.001
College or higher education (%)	13.5	17.0	18.8	18.2	17.0	<0.001
High perceived mental stress (%)	22.9	26.7	23.4	21.2	19.0	<0.001
**Women**						
No. of subjects	7106	7107	7106	7107	7106	
Median non-heme iron intake (mg/day)	3.81	5.91	6.96	7.98	9.46	
Mean age (SD) (years)	55.8 (10.0)	54.9 (9.8)	55.4 (9.8)	56.7 (9.7)	58.6 (9.7)	<0.001
Mean body mass index (kg/m^2^)	23.3 (3.2)	22.8 (3.0)	22.8 (3.0)	22.8 (3.0)	22.9 (3.0)	<0.001
Mean energy intake (SD) (kcal/day)	1404 (385)	1451 (379)	1459 (354)	1474 (343)	1399 (354)	<0.001
Mean dietary sodium intake (mg/day)	1636	1706	2046	2333	2587	<0.001
Current smokers (%)	6.6	4.7	3.9	3.4	3.5	<0.001
Mean alcohol intake (g/day of ethanol)	13.7	10.4	9.3	8.0	7.7	<0.001
History of hypertension (%)	23.5	19.2	19.1	20.2	20.5	<0.001
History of diabetes mellitus (%)	3.6	3.0	3.2	3.3	4.0	0.493
Sports time ≥3 hours/week (%)	7.5	8.6	9.2	10.0	11.3	<0.001
Walking time ≥30 minutes/day (%)	43.4	47.9	45.5	44.8	43.8	<0.001
College or higher education (%)	7.0	10.0	11.2	10.5	9.9	<0.001
High perceived mental stress (%)	19.2	21.4	20.7	18.6	16.7	<0.001
Menopause (%)	62.3	59.6	61.7	66.0	70.4	<0.001
Hormone-replacement therapy (%)	4.8	4.6	4.3	4.1	4.3	<0.001

As compared with subjects in the lowest quintile of total iron intake, those in the higher quintiles were older, less likely to be a current smoker, more likely to have high perceived mental stress, consumed less ethanol, and played sports more. History of hypertension was similar between sexes. Similar trends are shown in Tables 2 and 3. However, as compared with subjects in the lowest quintile of heme iron intake, both man and women in the higher quintiles had lower proportions of hypertension.

During 859 450 person-years of follow-up, we documented 2690 deaths (1343 men and 1347 women) from total CVD. Among men, 607 deaths were due to total stroke (which included 196 hemorrhagic strokes and 355 ischemic strokes), 311 to CHD, and 243 to myocardial infarction. Among women, there were 620 total stroke deaths (including 263 hemorrhagic strokes and 296 ischemic stroke), 246 CHD deaths, and 185 deaths due to myocardial infarction.

As shown in Table 4 , dietary intake of total iron showed a tendency to be associated with increased mortality from total and ischemic strokes among men in the age-adjusted model. These associations became more evident after further adjustment for known cardiovascular risk factors: the multivariable HRs for the highest versus lowest quintile of total iron intake were 1.43 (95% CI, 1.02–2.00; *P* for trend = 0.01) for total stroke and 1.27 (1.01–1.58; 0.02) for total CVD. Among women, dietary iron intake showed a tendency to be inversely associated with mortality from CHD, but the multivariable HR for the highest versus lowest quintile of total iron intake did not reach statistical significance.

**Table 4.** Sex-specific hazard ratios (95% CI) of mortality from stroke, coronary heart disease, heart failure, and total cardiovascular disease by quintile of total iron intake

	Quintiles of total iron intake					*P* for trend
	
	1 (low)	2	3	4	5 (high)	
**Men**						
Number of subjects	4616	4617	4617	4617	4616	
Person-years	65 666	66 308	67 818	67 305	65 423	
Total stroke						
*n*	79	93	103	145	187	
Age-adjusted HR	1.00	1.07 (0.79–1.45)	0.93 (0.70–1.25)	1.14 (0.87–1.50)	1.22 (0.94–1.59)	0.066
Multivariable HR^a^	1.00	1.11 (0.81–1.54)	0.99 (0.71–1.37)	1.26 (0.91–1.75)	1.43 (1.02–2.00)	0.009
Hemorrhagic stroke						
*n*	32	36	35	38	55	
Age-adjusted HR	1.00	1.05 (0.66–1.70)	0.89 (0.55–1.44)	0.89 (0.56–1.43)	1.16 (0.75–1.81)	0.567
Multivariable HR^a^	1.00	1.18 (0.71–1.96)	1.06 (0.62–1.82)	1.12 (0.64–1.96)	1.62 (0.92–2.85)	0.083
Ischemic stroke						
*n*	40	49	58	88	120	
Age-adjusted HR	1.00	1.11 (0.73–1.68)	0.97 (0.65–1.45)	1.23 (0.85–1.80)	1.34 (0.93–1.92)	0.046
Multivariable HR^a^	1.00	1.13 (0.72–1.76)	0.95 (0.60–1.49)	1.25 (0.80–1.96)	1.40 (0.89–2.21)	0.056
Coronary heart disease						
*n*	58	58	44	65	86	
Age-adjusted HR	1.00	0.91 (0.63–1.31)	0.56 (0.38–0.82)	0.73 (0.51–1.05)	0.83 (0.60–1.17)	0.346
Multivariable HR^a^	1.00	1.00 (0.67–1.47)	0.62 (0.40–0.96)	0.86 (0.56–1.32)	0.93 (0.60–1.45)	0.959
Myocardial infarction						
*n*	50	42	34	49	68	
Age-adjusted HR	1.00	0.77 (0.51–1.16)	0.51 (0.33–0.79)	0.66 (0.44–0.98)	0.80 (0.55–1.16)	0.381
Multivariable HR^a^	1.00	0.85 (0.54–1.32)	0.57 (0.35–0.93)	0.76 (0.47–1.23)	0.87 (0.53–1.41)	0.882
Total cardiovascular disease						
*n*	185	221	241	296	400	
Age-adjusted HR	1.00	1.08 (0.89–1.32)	0.94 (0.78–1.13)	1.00 (0.83–1.21)	1.14 (0.95–1.35)	0.161
Multivariable HR^a^	1.00	1.13 (0.92–1.39)	0.99 (0.80–1.23)	1.11 (0.89–1.38)	1.27 (1.01–1.58)	0.023
**Women**						
Number of subjects	7106	7107	7106	7107	7106	
Person-years	101 715	104 019	106 270	107 404	107 568	
Total stroke						
*n*	106	109	131	124	150	
Age-adjusted HR	1.00	1.04 (0.80–1.36)	1.07 (0.83–1.38)	0.84 (0.65–1.09)	0.88 (0.68–1.13)	0.100
Multivariable HR^a^	1.00	0.98 (0.74–1.30)	0.98 (0.74–1.30)	0.74 (0.55–0.99)	0.77 (0.57–1.04)	0.024
Hemorrhagic stroke						
*n*	49	46	62	49	57	
Age-adjusted HR	1.00	0.92 (0.62–1.38)	1.12 (0.77–1.62)	0.77 (0.52–1.15)	0.81 (0.55–1.19)	0.166
Multivariable HR^a^	1.00	0.93 (0.61–1.41)	1.11 (0.73–1.68)	0.75 (0.48–1.18)	0.78 (0.50–1.23)	0.162
Ischemic stroke						
*n*	46	51	59	62	78	
Age-adjusted HR	1.00	1.18 (0.79–1.75)	1.10 (0.75–1.62)	0.92 (0.63–1.35)	0.97 (0.63–1.40)	0.485
Multivariable HR^a^	1.00	1.04 (0.68–1.58)	0.92 (0.60–1.41)	0.74 (0.48–1.13)	0.78 (0.50–1.20)	0.107
Coronary heart disease						
*n*	49	43	43	54	57	
Age-adjusted HR	1.00	0.90 (0.60–1.36)	0.75 (0.50–1.13)	0.77 (0.52–1.13)	0.69 (0.47–1.01)	0.047
Multivariable HR^a^	1.00	0.98 (0.64–1.51)	0.91 (0.58–1.42)	0.96 (0.61–1.49)	0.89 (0.57–1.41)	0.643
Myocardial infarction						
*n*	39	33	24	39	50	
Age-adjusted HR	1.00	0.87 (0.55–1.38)	0.53 (0.32–0.88)	0.70 (0.45–1.09)	0.77 (0.50–1.17)	0.224
Multivariable HR^a^	1.00	0.97 (0.60–1.57)	0.64 (0.37–1.11)	0.92 (0.55–1.53)	1.04 (0.62–1.73)	0.745
Total cardiovascular disease						
*n*	233	232	259	269	354	
Age-adjusted HR	1.00	1.01 (0.84–1.22)	0.95 (0.80–1.14)	0.81 (0.68–0.97)	0.92 (0.78–1.08)	0.086
Multivariable HR^a^	1.00	1.01 (0.83–1.22)	0.97 (0.79–1.17)	0.82 (0.67–0.99)	0.94 (0.77–1.15)	0.301

HR: hazard ratio.

^a^Adjusted further for body mass index, smoking status, ethanol intake, history of hypertension, history of diabetes mellitus, sports time, walking time, educational status, perceived mental stress, dietary sodium intake, and, for women, menopausal status and hormone replacement therapy.

Table 5 shows the relationship between heme iron intake and mortality from cardiovascular disease in the multivariable model. Heme iron intake was inversely associated with mortality from myocardial infarction in men. The multivariable HR for the highest versus lowest quintile of heme iron intake was 0.59 (95% CI, 0.38–0.90; *P* for trend = 0.015). There was no significant association between heme iron intake and mortality from any cardiovascular disease endpoint in women.

**Table 5.** Hazard ratios (95% CI) of mortality from stroke, coronary heart disease, heart failure, and total cardiovascular disease according by quintile of heme iron intake

	Quintiles of heme iron intake					*P* for trend
	
	1 (low)	2	3	4	5 (high)	
**Men**						
Number of subjects	4616	4617	4617	4617	4616	
Person-years	67 446	66 998	67 081	66 607	64 390	
Total stroke						
*n*	153	111	108	102	133	
Multivariable HR^a^	1.00	0.93 (0.72–1.21)	0.95 (0.73–1.23)	0.84 (0.64–1.09)	1.04 (0.81–1.33)	0.833
Hemorrhagic stroke						
*n*	40	41	46	26	43	
Multivariable HR^a^	1.00	1.17 (0.71–1.75)	1.29 (0.83–2.01)	0.71 (0.42–1.18)	1.13 (0.72–1.78)	0.988
Ischemic stroke						
*n*	95	65	53	67	75	
Multivariable HR^a^	1.00	0.97 (0.70–1.35)	0.84 (0.59–1.19)	0.97 (0.70–1.35)	1.03 (0.70–1.35)	0.810
Coronary heart disease						
*n*	80	56	62	64	49	
Multivariable HR^a^	1.00	0.88 (0.62–1.26)	1.02 (0.72–1.44)	1.02 (0.72–1.45)	0.74 (0.51–1.07)	0.190
Myocardial infarction						
*n*	69	47	50	43	34	
Multivariable HR^a^	1.00	0.86 (0.58–1.26)	0.94 (0.64–1.38)	0.79 (0.53–1.18)	0.59 (0.38–0.90)	0.015
Total cardiovascular disease						
*n*	341	247	230	245	280	
Multivariable HR^a^	1.00	0.91 (0.77–1.08)	0.88 (0.74–1.05)	0.89 (0.75–1.06)	0.96 (0.81–1.14)	0.740
**Women**						
Number of subjects	7106	7106	7106	7106	7106	
Person-years	107 688	105 828	105 477	104 747	103 238	
Total stroke						
*n*	180	138	114	100	88	
Multivariable HR^a^	1.00	1.01 (0.80–1.27)	0.95 (0.75–1.22)	0.91 (0.70–1.17)	0.80 (0.61–1.04)	0.073
Hemorrhagic stroke						
*n*	72	57	51	42	41	
Multivariable HR^a^	1.00	0.97 (0.67–1.38)	0.94 (0.64–1.36)	0.81 (0.55–1.21)	0.80 (0.54–1.20)	0.208
Ischemic stroke						
*n*	88	74	48	48	38	
Multivariable HR^a^	1.00	1.15 (0.83–1.58)	0.88 (0.61–1.27)	0.99 (0.69–1.44)	0.77 (0.52–1.14)	0.156
Coronary heart disease						
*n*	74	49	33	41	49	
Multivariable HR^a^	1.00	0.88 (0.61–1.28)	0.70 (0.46–1.07)	0.99 (0.67–1.48)	1.18 (0.80–1.72)	0.352
Myocardial infarction						
*n*	58	35	23	28	41	
Multivariable HR^a^	1.00	0.81 (0.53–1.25)	0.63 (0.38–1.03)	0.87 (0.55–1.40)	1.25 (0.82–1.91)	0.281
Total cardiovascular disease						
*n*	386	287	236	217	221	
Multivariable HR^a^	1.00	0.97 (0.83–1.13)	0.91 (0.77–1.08)	0.93 (0.78–1.10)	0.94 (0.79–1.12)	0.405

HR: hazard ratio.

^a^Adjusted further for body mass index, smoking status, ethanol intake, history of hypertension, history of diabetes mellitus, sports time, walking time, educational status, perceived mental stress, dietary sodium intake, and, for women, menopausal status and hormone replacement therapy.

As shown in Table 6 , non-heme iron intake was associated with mortality from hemorrhagic stroke in men: the multivariable HR for the highest versus lowest quintile of non-heme iron intake was 1.72 (95% CI, 1.02–2.90; *P* for trend = 0.038). The corresponding HRs were 1.77 (0.95–3.31; 0.05) for intraparenchymal hemorrhage (no. of deaths = 141) and 1.74 (95% CI, 0.66–4.59; *P* for trend = 0.36) for subarachnoid hemorrhage (no. of deaths = 55). In women, non-heme iron was not associated with mortality from any cardiovascular disease endpoint.

**Table 6.** Hazard ratios (95% CI) of mortality from stroke, coronary heart disease, heart failure and total cardiovascular disease by quintile of non-heme iron intake

	Quintiles of non-heme iron intake					*P* for trend
	
	1 (low)	2	3	4	5 (high)	
**Men**						
Number of subjects	4616	4617	4617	4617	4616	
Person-years	65 974	66 161	67 062	67 666	65 660	
Total stroke						
*n*	117	82	91	138	179	
Multivariable HR^a^	1.00	0.93 (0.69–1.26)	0.85 (0.63–1.15)	1.01 (0.83–1.46)	1.19 (0.90–1.57)	0.088
Hemorrhagic stroke						
*n*	33	33	36	39	55	
Multivariable HR^a^	1.00	1.14 (0.69–1.90)	1.20 (0.72–2.00)	1.23 (0.73–2.08)	1.72 (1.02–2.90)	0.038
Ischemic stroke						
*n*	73	41	48	82	111	
Multivariable HR^a^	1.00	0.85 (0.57–1.29)	0.74 (0.50–1.11)	1.02 (0.71–1.46)	1.03 (0.72–1.48)	0.495
Coronary heart disease						
*n*	76	49	50	58	78	
Multivariable HR^a^	1.00	0.81 (0.56–1.19)	0.73 (0.49–1.08)	0.75 (0.51–1.11)	0.81 (0.55–1.18)	0.326
Myocardial infarction						
*n*	63	38	39	41	62	
Multivariable HR^a^	1.00	0.77 (0.50–1.19)	0.71 (0.46–1.09)	0.66 (0.42–1.02)	0.80 (0.52–1.22)	0.312
Total cardiovascular disease						
*n*	271	189	221	283	379	
Multivariable HR^a^	1.00	0.90 (0.74–1.10)	0.87 (0.72–1.06)	0.96 (0.80–1.17)	1.04 (0.86–1.26)	0.386
**Women**						
Number of subjects	7106	7106	7106	7106	7106	
Person-years	105 114	101 826	105 246	107 307	107 483	
Total stroke						
*n*	130	95	116	126	153	
Multivariable HR^a^	1.00	1.01 (0.77–1.34)	1.04 (0.79–1.37)	0.89 (0.67–1.17)	0.86 (0.65–1.12)	0.154
Hemorrhagic stroke						
*n*	52	43	59	51	58	
Multivariable HR^a^	1.00	1.08 (0.70–1.65)	1.33 (0.88–1.99)	0.97 (0.63–1.50)	0.94 (0.61–1.44)	0.579
Ischemic stroke						
*n*	62	41	50	64	79	
Multivariable HR^a^	1.00	0.92 (0.60–1.39)	0.88 (0.58–1.33)	0.82 (0.55–1.22)	0.76 (0.52–1.12)	0.155
Coronary heart disease						
*n*	58	41	37	53	57	
Multivariable HR^a^	1.00	0.95 (0.63–1.45)	0.80 (0.51–1.24)	0.96 (0.63–1.46)	0.85 (0.55–1.29)	0.491
Myocardial infarction						
*n*	45	32	22	36	50	
Multivariable HR^a^	1.00	0.95 (0.59–1.53)	0.61 (0.35–1.05)	0.87 (0.53–1.41)	0.98 (0.61–1.58)	0.999
Total cardiovascular disease						
*n*	271	217	230	275	354	
Multivariable HR^a^	1.00	1.09 (0.90–1.31)	1.00 (0.83–1.21)	0.96 (0.79–1.15)	0.99 (0.83–1.19)	0.661

HR: hazard ratio.

^a^Adjusted further for body mass index, smoking status, ethanol intake, history of hypertension, history of diabetes mellitus, sports time, walking time, educational status, perceived mental stress, dietary sodium intake, and, for women, menopausal status and hormone replacement therapy.

## DISCUSSION

In this large prospective cohort study with a median follow-up of 14.7 years, we found that greater dietary iron intake was associated with a higher risk of mortality from total stroke in Japanese men.

Several studies have examined the association between serum ferritin, an indicator of body iron stores, and stroke risk, but the results have been inconsistent. A prospective study of 11 471 Dutch postmenopausal women aged 49 to 70 years showed that higher serum ferritin concentrations were associated with increased risk of ischemic stroke.^7^ In that study, the multivariable risk ratio for the highest versus lowest tertile of serum ferritin concentration was 2.23 (95% CI, 1.05–4.73). However, a 17-year follow-up study in Australia, consisting of 1612 men and women aged 40 to 89 years, reported that serum ferritin was not associated with risk of total stroke: the HRs for the highest versus lowest tertile were 1.71 (0.72–1.49) for men, 0.99 (0.38–2.59) for women, and 1.43 (0.78–2.64) for all subjects.^12^

There is little evidence regarding the association of dietary iron intake with stroke risk. A recent case-control study comprising 374 ischemic stroke cases and 464 hospital-based controls showed that higher iron intake was associated with greater risk of ischemic stroke: the odds ratio for the highest (≥161 mg/wk) versus lowest (≤100 mg/wk) tertile was 2.43 (95% CI, 1.06–5.58; *P* for trend = 0.03). However, that association became insignificant after adjustment for other CVD risk factors.^14^ To our knowledge, our study is the first prospective study to show a positive association between dietary iron intake and stroke mortality in men.

The present study showed no significant association between dietary iron intake and mortality from CHD or myocardial infarction in either sex. A meta-analysis of 12 prospective studies that were conducted before 1998 and involved 7800 CHD cases also showed no association between iron status and CHD risk.^8^ A comparison of subjects in the top versus bottom quintile of baseline variables revealed nonsignificant risk ratios for total iron-binding capacity: the combined risk ratio was 1.0 (95% CI, 0.7–1.5), and the risk ratio was 0.8 (0.7–1.0) for serum iron and 0.8 (0.7–1.1) for total dietary iron.^8^ As for serum ferritin levels, in 5 studies involving 570 cases of CHD, a comparison of individuals with baseline values of 200 mg/L or higher versus those with values less than 200 mg/L yielded a combined risk ratio of 1.0 (95% CI, 0.8–1.3). As for transferrin saturation levels, in 5 studies involving 6194 cases of CHD, a comparison of individuals in the top versus bottom tertile of the baseline measurement yielded a combined risk ratio of 0.9 (95% CI, 0.7–1.1).^8^ In contrast, a 13-year follow-up study consisting of 4237 residents aged 40 to 74 years showed that serum iron was inversely associated with risk of myocardial infarction: the multivariable relative risk associated with an increase of 5.4 µmol/L in serum iron concentration was 0.82 in women (95% CI, 0.70–0.95) and 0.92 (0.85–1.00) in men.^17^

Because of its high absorption rate, we separated the analysis of dietary heme iron from that of total dietary iron in our investigation of associations with cardiovascular disease mortality. In men with repletion iron stores, 26% of dietary heme iron was absorbed, while only 2.5% of non-heme iron was absorbed.^23^ However, only a few studies have focused on the relationship between heme iron intake and CVD. The Health Professionals Follow-up Study found that the risk of fatal CHD or nonfatal myocardial infarction was higher among men in the top quintile of heme iron intake as compared with those in the lowest quintile: the multivariable relative risk was 1.42 (95% CI, 1.02–1.98; *P* for trend = 0.02).^15^ The European Prospective Investigation into Cancer and Nutrition also showed that higher dietary heme iron intake was associated with increased CHD risk among women.^16^ In that study, the multivariable HR for CHD development among women in the highest versus the lowest quintile of heme iron intake was 1.65 (1.07–2.53; 0.02).

In contrast, the present study showed an inverse association between heme iron intake and mortality from myocardial infarction among men. The reasons for these contradictory trends are unknown. However, 1 reason might be that dietary heme iron intake among Japanese is lower than that among Western people. Mean heme iron intake in the present study was 0.25 mg/day for men and 0.24 mg/day for women, which was much lower than that for European women (1.81 mg/day), even when underestimation of our values was taken into account.^16^ Dietary heme iron intake in Japanese adults may not be high enough to develop high body iron stores and increase CVD risk. Non-heme iron was positively associated with mortality from hemorrhagic stroke in men.

We found no association between dietary iron intake and cardiovascular disease mortality among women. Two studies of European and American women also showed no associations,^17^^,^^24^ but 1 study of European women showed a positive association between heme iron intake and CHD risk.^16^ A reason for the lack of association is that iron stores are lower in women than in men.^3^

The role of iron in the development of stroke and CVD is not established. Iron catalyzes production of highly reactive hydroxyl free radicals and promotes LDL oxidation, leading to atherosclerosis.^25^ However, this mechanism is unlikely to explain our findings because the major pathologic process for stroke among Japanese is arteriolosclerosis (in which oxidized LDL has only a limited role) rather than atherosclerosis.^26^ Iron-dependent oxidative stress may lead to cell death, ie, necrosis and apoptosis,^27^ which may partly explain the excess risk of hemorrhagic stroke (intraparenchymal or subarachnoid hemorrhage) associated with high non-heme iron intake, as the basic pathologic process of hemorrhagic stroke is necrosis or apoptosis of vascular wall cells in cerebral arteries.^28^^,^^29^ However, our finding that high iron intake was associated with an increased risk of hemorrhagic stroke among men could have been observed by chance and thus requires further investigation. Free radicals that form during brain ischemia and reperfusion may induce vasodilatation and increase blood–brain barrier permeability to macromolecules, which leads to brain damage.^30^ Brain damage is promoted by iron-foaming hydroxyl free radicals,^25^ which are associated with poor stroke prognosis.^30^ An experimental study of rats found that ischemic brain injury was reduced by free radical scavengers.^31^ These pathologic processes may in part explain the excess risk of mortality from stroke.

The strengths of the present study include its large population-based sampling from throughout Japan and its prospective design. In addition, the measurement of exposure variable covariates and outcomes was standardized through the use of a uniform questionnaire and surveillance protocol. However, the study also had limitations. First, only approximately 53% of potential participants responded to the FFQ. Respondents were 3 years younger, more educated, and had more perceived mental stress than did nonresponders. Thus, we adjusted these variables in our examination of the associations between iron intake and cardiovascular disease mortality. Second, dietary iron intake may not accurately reflect total body iron stores, because gastrointestinal absorption varies with body iron status, age, and inflammation status, among other factors.^18^ Third, the exposure data were collected only once, at the baseline survey, and the subjects were followed over time. It is possible that the subjects changed their diets during the follow-up period, which could have weakened the true association between dietary iron intake and CVD mortality.

In summary, the present study showed that a high dietary intake of iron was associated with increased mortality from total stroke and total CVD in Japanese men. These findings lend support to the hypothesis that excess iron intake increases CVD risk.

## ONLINE ONLY MATERIALS

The Japanese-language abstract for articles can be accessed by clicking on the tab labeled Supplementary materials at the journal website http://dx.doi.org/10.2188/jea.JE20120006.

Abstract in Japanese.
