# Supplementary material for: Associations of Dietary Iron Intake With Mortality From Cardiovascular Disease: The JACC Study
Source: J Epidemiol. 2012 Nov 5;22(6):484–93. doi: 10.2188/jea.JE20120006 (PMC3798559; doi:10.2188/jea.JE20120006)
Supplement: Abstract in Japanese. [file je-22-484-s001.pdf]

# JACC Studyにおける食事からの鉄分摂取と循環器疾患の関連

Associations of dietary iron intake with mortality from cardiovascular disease: the JACC study

[1]Wen Zhang、[1]磯博康、[1]大平哲也、[2]伊達ちぐさ、[3]田邊　直仁、[4]菊地 正悟、[4]玉腰暁子、JACC Study Group

大阪大学大学院医学研究科社会環境医学講座公衆衛生学[1]、兵庫県立大学環境人間学部[2]、新潟県立大学人間生活学部[3]、愛知医科大学医学部公衆衛生[4]

[目的]本研究は、日本人を対象とし、大規模縦断研究で鉄の摂取が循環器疾患死亡との関連を研究することを目的とする。

[方法ならびに成績]本研究は、文部科学省科学研究費がん特定領域大規模コホート研究(JACC Study)の2006年までのデータに基づいて、1988年から1990年のベースライン時に脳卒中、CHDの既往歴がなく、FFQに記入した40-79歳の男性23,083、女性35,532名を研究対象とした。鉄の摂取量について、男女別に残差法によりエネルギーを調整して男女別に五分位で分析を行った。循環器疾患死亡の相対危険度を多変量調整したCox比例ハザードモデルにより算出した。

追跡期間中2,690人（男性1,343人、女性1,347人）、そのうち、脳卒中1,227人（男性607人、女性620人）、脳梗塞651人（男性355人、女性296人)、脳出血459人（男性196人、女性263人),虚血性心疾患557人(男性311人、女性246人)が、循環器疾患で死亡した。男性において、鉄の摂取の最も低い群に対して最高群の多変量調整の全脳卒中と全循環器疾患の死亡リスクはそれぞれ1.43 (95%CI,1.02-2.00, P for trend=0.009)、1.27(95%CI,1.01-1.58, P for trend=0.023)であった。女性において、鉄の摂取と循環器疾患の死亡リスクの間に有意な関連は認められなかった。

[総括]日本人の男性において、鉄の摂取と全脳卒中および全循環器疾患の死亡リスクの間に正の関連がある。

キーワード：食事からの鉄、死亡、脳卒中、虚血性心疾患、循環器疾患、フォローアップ研究
